# A novel cost-effective technology to convert sucrose and homocelluloses in sweet sorghum stalks into ethanol

**DOI:** 10.1186/1754-6834-6-174

**Published:** 2013-11-29

**Authors:** Jihong Li, Shizhong Li, Bing Han, Menghui Yu, Guangming Li, Yan Jiang

**Affiliations:** 1 Institute of Nuclear and New Energy Technology, Tsinghua University, Beijing 100084, People’s Republic of China; 2 Beijing Engineering Research Center of Biofuels, MOST-USDA joint research center for biofuels, Beijing 100084, People’s Republic of China

**Keywords:** Sweet sorghum stalks, Alkaline pretreatment, Solid-state fermentation, Bioethanol

## Abstract

**Background:**

Sweet sorghum is regarded as a very promising energy crop for ethanol production because it not only supplies grain and sugar, but also offers lignocellulosic resource. Cost-competitive ethanol production requires bioconversion of all carbohydrates in stalks including of both sucrose and lignocellulose hydrolyzed into fermentable sugars. However, it is still a main challenge to reduce ethanol production cost and improve feasibility of industrial application. An integration of the different operations within the whole process is a potential solution.

**Results:**

An integrated process combined advanced solid-state fermentation technology (ASSF) and alkaline pretreatment was presented in this work. Soluble sugars in sweet sorghum stalks were firstly converted into ethanol by ASSF using crushed stalks directly. Then, the operation combining ethanol distillation and alkaline pretreatment was performed in one distillation-reactor simultaneously. The corresponding investigation indicated that the addition of alkali did not affect the ethanol recovery. The effect of three alkalis, NaOH, KOH and Ca(OH)_2_ on pretreatment were investigated. The results indicated the delignification of lignocellulose by NaOH and KOH was more significant than that by Ca(OH)_2_, and the highest removal of xylan was caused by NaOH. Moreover, an optimized alkali loading of 10% (w/w DM) NaOH was determined. Under this favorable pretreatment condition, enzymatic hydrolysis of sweet sorghum bagasse following pretreatment was investigated. 92.0% of glucan and 53.3% of xylan conversion were obtained at enzyme loading of 10 FPU/g glucan. The fermentation of hydrolyzed slurry was performed using an engineered stain, Zymomonas mobilis TSH-01. A mass balance of the overall process was calculated, and 91.9 kg was achieved from one tonne of fresh sweet sorghum stalk.

**Conclusions:**

A low energy-consumption integrated technology for ethanol production from sweet sorghum stalks was presented in this work. Energy consumption for raw materials preparation and pretreatment were reduced or avoided in our process. Based on this technology, the recalcitrance of lignocellulose was destructed via a cost-efficient process and all sugars in sweet sorghum stalks lignocellulose were hydrolysed into fermentable sugars. Bioconversion of fermentable sugars released from sweet sorghum bagasse into different products except ethanol, such as butanol, biogas, and chemicals was feasible to operate under low energy-consumption conditions.

## Background

Increased fossil fuel consumption has resulted in a series of social and environmental problems, such as the crisis of oil, global climate change and the emission of greenhouse gas. Sustainable and clean renewable energy as an alternative to fossil fuels has attracted extensive attention worldwide. Among various renewable energies, bioethanol is an important renewable liquid fuel due to its high octane number and heat of vaporization. Bioethanol is also less volatile than gasoline, has a lower photochemical reactivity in the atmosphere, and smog formation from emissions of pure ethanol can be less than from gasoline [1].

Sweet sorghum is a high photosynthetic-efficiency energy crop with high biomass (20 to 30 dry tonnes/ha) and sugar-yielding (16 to 18% fermentable sugar in juice) [2]. It is also the only crop that provides grain and sugar, and a lignocellulosic biomass resource. Sweet sorghum has several primary advantages, such as (1) its adaptability to diverse climate zones and soil conditions (salinity, alkalinity and drought); (2) low requirement of fertilizers; (3) high water-usage efficiency compared with more conventional crops (1/3 of sugarcane and 1/2 of corn), and (4) short growth period (3 to 5 months) [3]. Based on these advantages, sweet sorghum can be planted on marginal lands. It will avoid competing for land against other cultures that are used for food production [4]. For these reasons, sweet sorghum has been regarded as an alcohol fuel crop with a promising future [5-7]. In fact, ethanol production from non-structural carbohydrates in sweet sorghum stalks is not difficult. There are two major kinds of technology to convert fermentable sugars to ethanol from sweet sorghum, one is liquid fermentation [4,8,9], the other is solid-state fermentation [10-12]. However, because there are approximately equal quantities of soluble and insoluble carbohydrates in sweet sorghum stalks [13], the major challenge for large-scale application of bioethanol production from sweet sorghum is how to deal with these lignocellulosic fractions (usually called bagasse). Cost-competitive ethanol production from sweet sorghum requires the bioconversion of all carbohydrates, including that of the sugar and lignocellulosic fraction, into ethanol.

Due to the recalcitrant nature of lignocellulosic materials, efficient bioconversion of sweet sorghum bagasse requires effective pretreatment to liberate cellulose from its physical seal and open up its crystalline structure before enzymatic hydrolysis can take place [14]. Although a range of chemical, physical and biological processes have been configured to release structural sugars from lignocellulose, they have to face the challenges of cost, infrastructure needs and technological breakthroughs [15]. An ideal pretreatment should have features as follows: (1) rendering high accessibility of biomass substrates to cellulases; (2) low capital and operational cost; (3) minimized size reduction of raw materials, and (4) producing low amounts of inhibitors to the enzymes and the fermentative microorganisms [16]. Compared to other pretreatment technologies, alkaline pretreatment processes generally utilize lower temperatures, pressures and residence times, and produce lower concentration of inhibitors [17]. Sodium hydroxide, potassium hydroxide and lime are usually used as an alkali reagent. The key role of alkaline is to partially remove lignin and hemicellulose in the biomass by disrupting the ester bonds cross-linking between lignin and xylan, thereby increasing the porosity of the biomass and resulting in cellulose and hemicellulose enriched fractions [18-20]. Enzymatic hydrolysis of sweet sorghum bagasse has been studied to some extent, and high enzymatic digestibility of sweet sorghum bagasse has also been reported [19-23]. However, the ethanol production cost is still high due to the complexity of the normal technology.

In the present study, a low energy-consumption and cost-efficient integrated process combining advanced solid-state fermentation technology (ASSF), alkaline pretreatment and C5-C6 co-fermentation in a whole process was configured. The effects of three alkalis, sodium hydroxide (NaOH), potassium hydroxide (KOH) and calcium hydroxide (Ca(OH)_2_) on the ethanol recovery, pretreatment and enzymatic digestibility of sweet sorghum bagasse were investigated. In order to study the total ethanol yield of the overall process, C5-C6 co-fermentation of hydrolysed slurry was performed using an engineer strain *Zymomonas mobilis* (*Z. mobilis*) *TSH-01*.

## Results and discussion

### Novel process flow of ethanol production from sweet sorghum stalks

Sweet sorghum shows a potential for ethanol production because its stalk is rich in both non-structural carbohydrates (sucrose, glucose and fructose) and structural carbohydrates (cellulose and hemicellulose) [20]. Cost-competitive ethanol production from sweet sorghum is challenged by the bioconversion of all carbohydrates from sugar and lignocellulose fractions into ethanol. The extraction of juice from the stalks is normally applied prior to pretreatment to prevent soluble sugar degradation. However, the squeezing operation needs high energy consumption. ASSF was studied in our previous work [21], and a demonstrated plant has been built up in Inner Mongolia province, China. The research on ASSF technology demonstrated that ASSF is a cost-efficient process, which can convert non-structural sugars to ethanol by anaerobic fermentation using the crushed sweet sorghum stalks directly in a rotatory drum fermenter [6,11]. After fermentation, almost all the non-structural sugars were consumed. The ethanol produced in the solid state fermentation step remained in the fermented bagasse. The ethanol separation was achieved by heating this fermented bagasse with low-pressure steam in a distillation stripper. In our ASSF technology, ethanol distillation from fermented bagasse was carried out at approximately 100°C, which is the temperature required for alkaline pretreatment. Therefore, the implementation of alkaline pretreatment is feasible, provided that the alkali does not negatively influence the distillation process. After this special distillation operation with alkali, the recalcitrant structure of sweet sorghum bagasse was disrupted.

**Figure 1:**
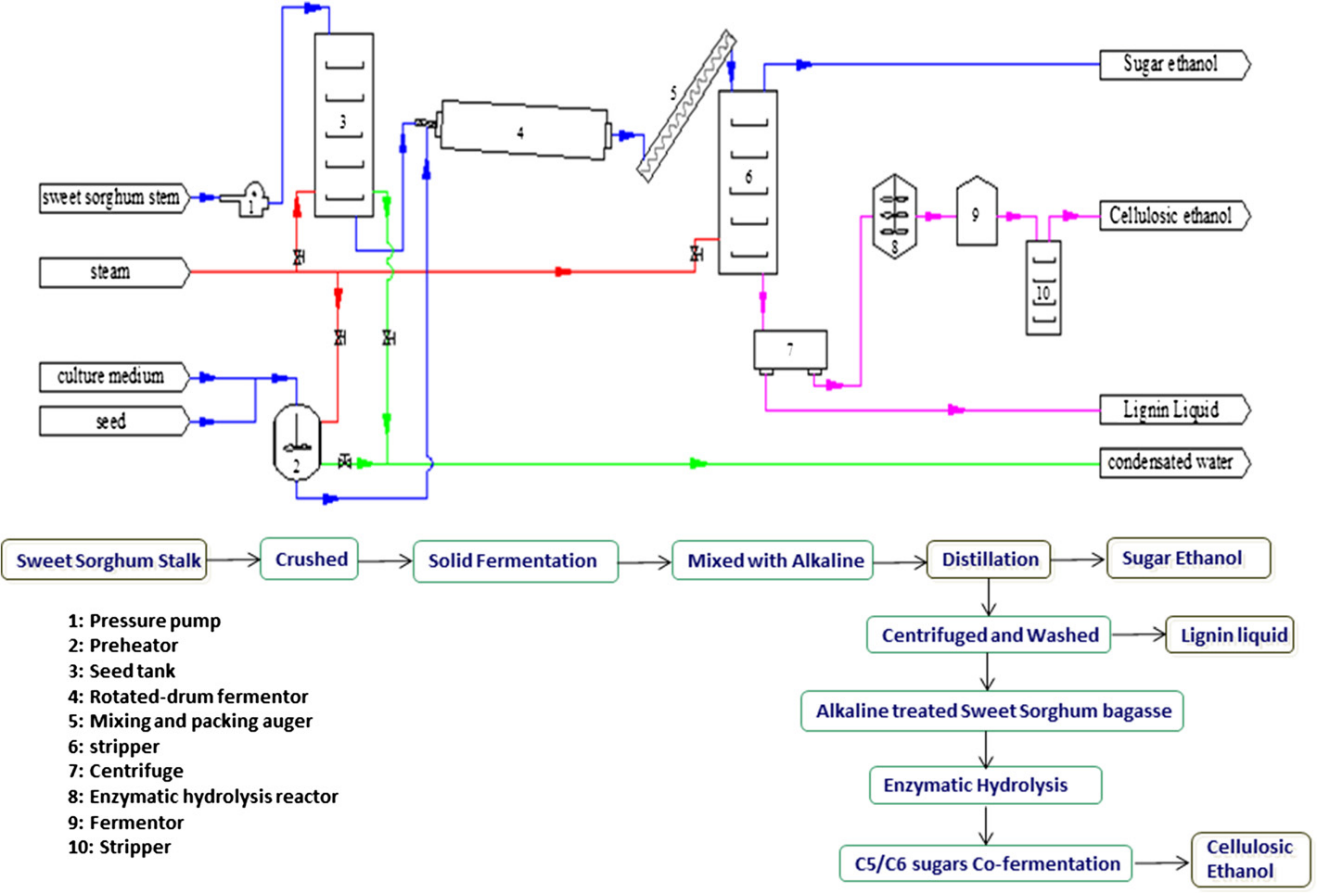
Process flow scheme of the novel cost-efficient integrated processes for ethanol production from sweet sorghum stalks.

The process flow schematic is shown in Figure 1. The fresh sweet sorghum was crushed by a pulverizer into particles 1 to 2 mm in diameter and 3 to 20 mm in length. Then the crushed sweet sorghum stalks, completely blended with 10% (v/w) of *Saccharomyces cerevisiae* TSH1 seed culture (about 25 g/L, dry weight), were added in a rotatory drum fermenter. The solid-state fermentation was performed for 24 h at 30°C with a rotary speed of 0.5 rpm. After the fermentation finished, the fermented bagasse containing ethanol was completely mixed with a certain volume of concentrated alkali solution. The fermented bagasse with alkali was transferred into a distillation stripper. The sugar-based ethanol remaining in the fermented bagasse was separated and collected by distillation. After distillation with alkali, the black liquor fraction, rich in lignin, was removed by centrifugation and the residual solids were washed with water, followed byfurther enzymatic hydrolyzation by a commercial cellulase at a 15% (w/w) solid loading. After 72 h enzymatic hydrolysis, the enzymatic slurry was anaerobically fermented using an engineered stain of *Z. mobilis* TSH-01. The cellulosic ethanol was separated from the fermentation broth.

From Figure 1, it is obvious that the integrated process retains all the advantages of solid-state fermentation technology, such as lower energy consumption for biomass material preparation and less waste water. Moreover, the equipment and the extra energy and time consumption for pretreatment was avoided by combining distillation and alkaline pretreatment in one step. Compared with ethanol production technology using sweet sorghum bagasse (obtained after extraction of juice from sweet sorghum stalks), this integrated technology significantly reduced energy consumption and the investment of infrastructure needs of pretreatment. Moreover, alkaline-pretreated bagasse partially retained hemicellulose, increasing the potential fermentable sugars compared to acid-based pretreatments.

### Influence of alkali in sugar-based ethanol distillation

**Figure 2:**
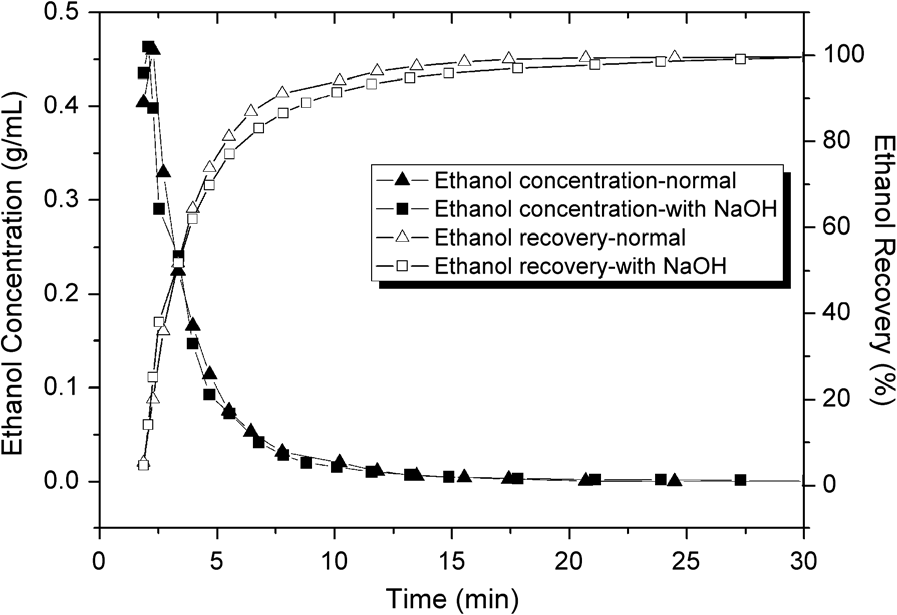
**Dynamic ethanol distillation profile of fermented sweet sorghum bagasse treated with 10% (w/w dry mass) sodium hydroxide.** NaOH, sodium hydroxide.

In order to study the influence of alkali in ethanol distillation, an ethanol distillation experiment was carried out with addition of NaOH. The ethanol distillation rate and ethanol recovery yield were investigated, and the results are shown in Figure 2 (the fermented bagasse without NaOH as a control).

The dynamic ethanol concentration profile obtained from the fermented bagasse with 10% (w/w dry mass (DM)) NaOH was similar to the control. In the presence of NaOH, the ethanol recovery rate was slightly lower than that without alkali. It took 24 minutes to obtain 99% of ethanol-recovery yield in the presence of NaOH, and 17 minutes in the absence of alkali. The ethanol content and recovery yield of fermented sweet sorghum bagasse is listed in Table 1. The results show that the ethanol content of fermented bagasse slightly decreased from 6.01 to 5.69 g per 100 g wet bagasse after mixing with NaOH, resulting from the release of ethanol during the mix operation. The loss of ethanol was about 3.4%, attributed to the mixed operation by hand in an open vessel in the laboratory. In practice, the loss of ethanol can be avoided by conducting the mixing operation in closed equipment. The ethanol recovery yield of bagasse with NaOH is 99.5%, equaling that of the control. This result demonstrated that ethanol-recovery yield was not affected by addition of alkali.

**Table 1:** Ethanol distillation of the fermented sweet sorghum bagasse

	**Normal distillation**		**Distillation with 10% (w/w DM) NaOH**	
	**Before**	**After**	**Before**	**After**
Sample mass for distillation (g)	4000	4962	4076	4828
Ethanol content in the sample (g/100 g WM^a^)	6.01	0.02	5.69	0.02
Ethanol in sample (g)	240.6	0.9	231.9	0.9
Recovered ethanol^b^ (g)		239.0		230.8
Ethanol recovery (%)		99.5		99.5

^a^WM represents the wet mass of sweet sorghum bagasse; ^b^The mass of recovered ethanol is calculated by the concentration (g/mL) of the distillate multiplied by the volume (mL) of the distillate. DM, dry mass.

### Influence of alkali loading in the composition of sweet sorghum bagasse

NaOH, KOH, ammonia and lime are alkali reagents commonly investigated in alkaline pretreatment of lignocellulosic biomass. Due to the volatility of ammonia, it is released quickly at 100°C, so it cannot react completely with lignocellulosic substrates during the ethanol distillation. For this reason, ammonia was excluded from our work. The influence of other three alkali reagents in pretreatment was investigated by preliminary distillation experiments due to the limitation of the available amounts of fermented sweet sorghum bagasse. The pretreatment temperature was fixed at 100°C by ethanol distillation. In addition, to achieve high ethanol-recovery yield, distillation should be carried out for more than 24 minutes to achieve ethanol recovery yield of 99%. Efficient alkaline pretreatment time of sweet sorghum bagasse has been reported to be in the range of 30 minutes to 100 h [18,19,22]. To balance the requirement of distillation with pretreatment, the distillation duration was set at 30 minutes. The intensity of pretreatment increased with increasing alkali loading from 0.83 to 6.67 mmol/g of dry biomass. The alkali loading was commonly expressed in term of g/g biomass in the study in which only one alkali was investigated. However, the stoichiometric ratio was not shown directly using this unit when there were several different alkalis, so the alkali loading in this work was expressed in terms of mmol/g of dry biomass, which refers to the ratio of the amount of alkali to dry weight of sweet sorghum bagasse.

Table 2 summarizes the solid recovery yield and the compositions of sweet sorghum bagasse following pretreatment with different alkali loading.

**Table 2:** Sweet sorghum bagasse recovered after distillation combined with alkaline pretreatment and main composition

**Alkali**	**Alkali loading (mmol/g dry biomass)**	**Solid recovery (%)**	**Composition (%)**		
			**Glucan**	**Xylan**	**Lignin**
Untreated	NA	NA	30.33 ± 0.34	20.93 ± 0.23	20.46 ± 0.64
NaOH	0.83	72.8 ± 1.3	41.22 ± 0.12	28.94 ± 0.78	15.93 ± 0.27
NaOH	1.67	61.8 ± 2.4	48.62 ± 0.21	39.07 ± 0.56	13.62 ± 0.43
NaOH	2.50	57.9 ± 1.7	51.31 ± 0.75	32.26 ± 0.37	11.21 ± 0.29
NaOH	3.33	51.8 ± 3.9	57.25 ± 0.35	32.38 ± 0.74	8.21 ± 0.25
NaOH	4.17	51.3 ± 3.3	57.69 ± 0.55	30.81 ± 0.63	7.34 ± 0.36
NaOH	5.00	49.3 ± 2.2	60.04 ± 0.81	29.34 ± 0.39	7.27 ± 0.20
NaOH	6.67	47.7 ± 0.9	61.63 ± 0.22	27.55 ± 0.49	7.86 ± 0.83
KOH	0.83	71.5 ± 1.8	42.01 ± 0.33	26.34 ± 0.52	16.94 ± 0.31
KOH	1.67	62.8 ± 3.1	47.84 ± 0.51	29.10 ± 0.16	13.23 ± 0.58
KOH	2.50	60.7 ± 1.6	49.28 ± 0.73	29.28 ± 0.63	9.92 ± 0.56
KOH	3.33	58.2 ± 1.2	51.07 ± 0.23	28.62 ± 0.04	8.01 ± 0.72
KOH	4.17	51.9 ± 2.4	57.10 ± 0.95	30.02 ± 0.77	6.50 ± 0.25
KOH	5.00	51.5 ± 0.7	57.55 ± 0.64	29.43 ± 0.19	7.02 ± 0.28
KOH	6.67	50.5 ± 2.1	57.97 ± 0.63	26.97 ± 0.83	7.26 ± 0.46
Ca(OH)_2_	0.83	78.3 ± 1.2	38.33 ± 0.76	23.89 ± 0.36	16.72 ± 0.39
Ca(OH)_2_	1.67	81.7 ± 0.8	36.73 ± 0.42	23.18 ± 0.73	15.98 ± 0.32
Ca(OH)_2_	2.50	68.7 ± 1.3	43.59 ± 0.33	26.55 ± 0.53	13.64 ± 0.17
Ca(OH)_2_	3.33	69.2 ± 0.5	42.98 ± 0.08	26.85 ± 0.71	15.05 ± 0.31
Ca(OH)_2_	4.17	70.7 ± 2.1	41.95 ± 0.55	26.20 ± 0.81	14.52 ± 0.48
Ca(OH)_2_	5.00	71.3 ± 1.7	41.57 ± 0.18	25.82 ± 0.31	13.18 ± 0.17
Ca(OH)_2_	6.67	69.5 ± 1.4	42.14 ± 0.38	26.13 ± 0.75	13.51 ± 0.42

NaOH, sodium hydroxide; KOH, potassium hydroxide; Ca(OH)_2_, calcium hydroxide; NA, not applicable. All the data are shown as mean ± SD.

**Figure 3:**
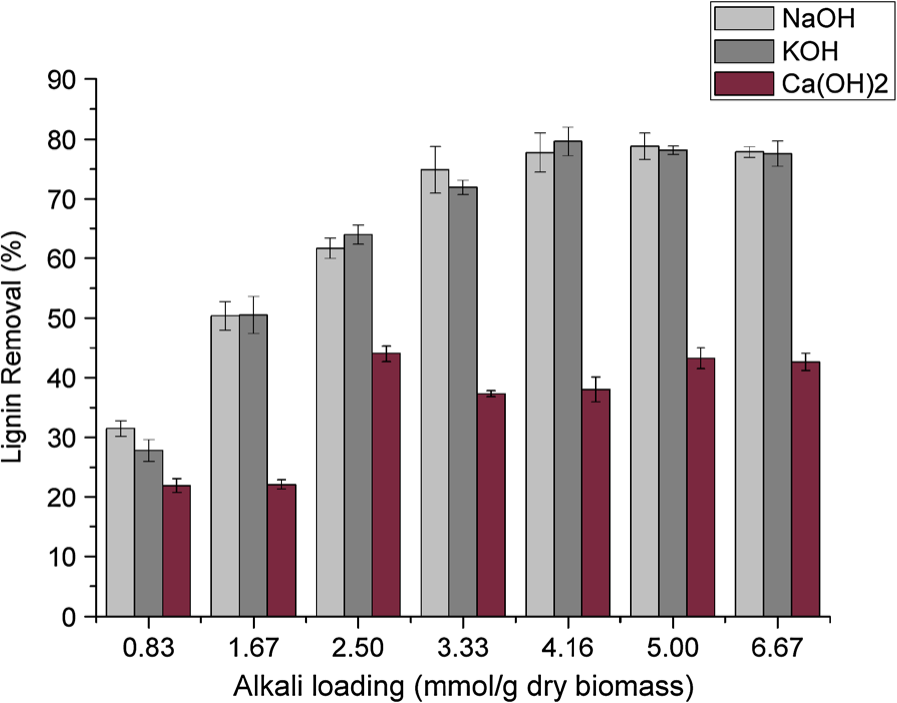
**Lignin removal of sweet sorghum bagasse following various alkali pretreatments.** NaOH, sodium hydroxide; KOH, potassium hydroxide; Ca(OH)_2_, calcium hydroxide.

**Figure 4:**
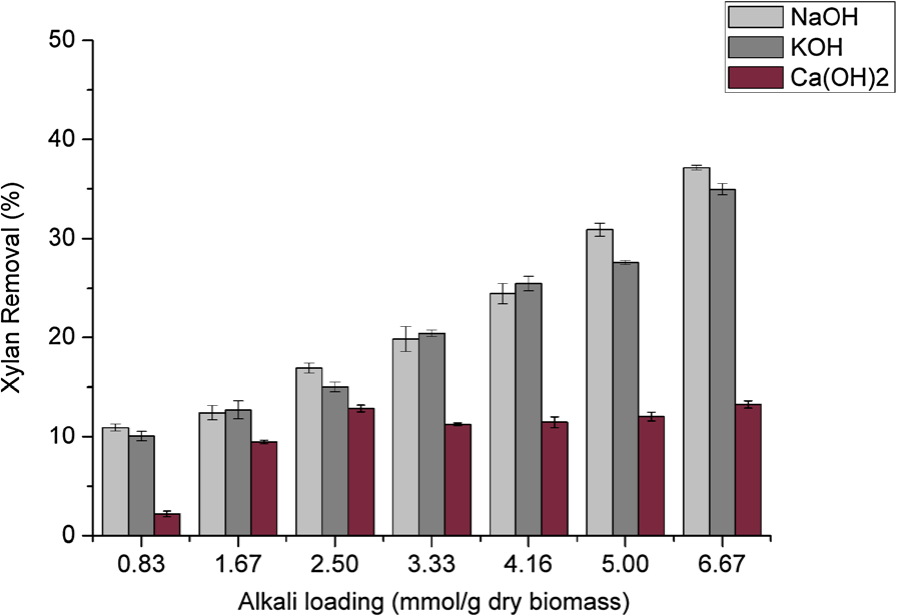
**Xylan removal of sweet sorghum bagasse following various alkali pretreatments.** NaOH, sodium hydroxide; KOH, potassium hydroxide; Ca(OH)_2_, calcium hydroxide.

As expected, lime performed worse than NaOH and KOH. During alkaline pretreatment, the cleavage of hydrolysable linkages, such as α- and β-aryl ethers in lignin and glycosidic bonds in carbohydrates, constitute the primary reactions that lead to the dissolution of lignin and carbohydrate with lower alkali stability [23]. Removal of hemicellulose and lignin, however, varied significantly depending on the pretreatment conditions (Figure 3 and Figure 4). The efficiency of delignification of the strong alkalis, NaOH and KOH, was significantly better than that of the weak alkali, Ca(OH)_2_. With increased loading of strong alkalis, the lignin removal increased until the alkali loading of 2.5 mmol/g of dry biomass; above this value the lignin removal did not increase significantly. The compositional analysis showed that almost all the lignin contents of bagasse following alkaline pretreatment were less than 10% when the loading of strong alkali of more than 2.5 mmol/g of dry biomass was used. Figure 3 shows that more than 70% of lignin was removed when the strong alkali loading was more than 3.33 mmol/g of dry biomass, whereas the highest lignin removal of bagasse treated with Ca(OH)_2_ was only 43.97% with loading of 2.5 mmol/g of dry biomass. Moreover, the results of compositional analysis showed that the lignin contents of bagasse were all more than 13% after Ca(OH)_2_ pretreatment. The result was in accordance with that of sugarcane bagasse treated with 0.40 g/g of lime at 90°C for 53.1 h, which resulted from the short pretreatment time [24]. Hence, pretreatment time is a crucial factor affecting the efficiency of lime pretreatment.

Cellulose was difficult to degrade under the alkaline condition [25], so the recovery yield of cellulose was more than 95% for all the samples following pretreatment. By increasing the intensity of the pretreatment, the cellulose content of bagasse increased gradually due to the removal of hemicellulose and lignin until the loading of 3.33 mmol/g of dry biomass. In accordance with the tendency of delignification, the cellulose content did not further increase significantly after the alkali loading used in the pretreatment exceeded 3.33 mmol/g of dry biomass. Ca(OH)_2_ pretreatment appeared to have weak ability to increase the cellulose content because the pretreatment time was too short. The cellulose content of bagasse treated with Ca(OH)_2_ varied from 38.33 to 42.98%, whereas that of bagasse treated with strong alkalis varied from 41.22 to 61.63% for NaOH, and from 42.01 to 57.97% for KOH.

The results of xylan removal are shown in Figure 4. With increasing concentration of strong alkalis, the removal of xylan increased linearly. Moreover, compared with KOH the hemicellulose had higher solubility in NaOH solution. The greatest xylan removal of 37.16% was caused by treatment with NaOH of 6.67 mmol/g of dry biomass, whereas it was 34.94% under KOH pretreatment. Compared with cellulose, the xylan content of bagasse following strong alkali pretreatment increased first to reach a peak, and then decreased gradually. The peak value occurred at alkali loading of 1.67 and 4.16 mmol/g of dry biomass for NaOH and KOH, respectively. This result was attributed to more hemicellulose dissolving in the alkaline solution at high alkali concentration. In contrast, lime has poor ability to dissolve the hemicellulose, and only less than 14% of xylan was removed from the bagasse under our process condition. Similar to lignin removal, the results demonstrated that to achieve the desirable pretreatment efficiency, lime needed more pretreatment time due to its low reactivity.

### Pretreatment efficiency by enzymatic digestibility

**Figure 5:**
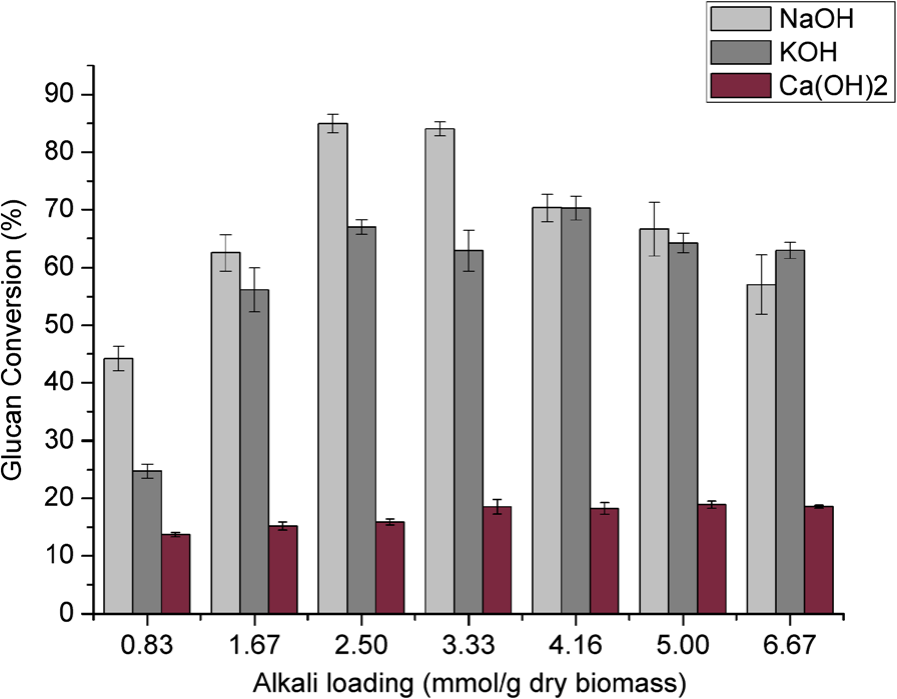
**Enzymatic hydrolysis of sweet sorghum bagasse following various alkali pretreatments.** NaOH, sodium hydroxide; KOH, potassium hydroxide; Ca(OH)_2_, calcium hydroxide.

The glucan conversions obtained during enzymatic hydrolysis of bagasse pretreated with different alkali loading are shown in Figure 5. With increasing loading dose of strong alkalis (NaOH and KOH), the glucan conversion of sweet sorghum bagasse following pretreatment increased first to reach a peak, and then decreased slightly. The peak value of 84.96% was achieved by NaOH loading of 2.5 mmol/g of dry biomass, and 70.25% peak value was achieved by KOH loading of 4.16 mmol/g of dry biomass. Compared with strong alkalis, the highest glucan conversion of bagasse treated with lime was only 18.87%. This result was attributed to low removal of lignin and hemicellulose. In addition, enzymatic activity was affected by the high pH value of the hydrolysis solution, which resulted from the considerable residual calcium hydroxide after pretreatment due to the low solubility of calcium hydroxide.

Alkalis help to reduce recalcitrance of biomass through saponification of hemicellulose acetyl and lignin-carbohydrate complex linkages [26,27]. As reported by Chang and Holtzapple, an effective lignocellulose treatment process should remove all the acetyl groups and reduce the lignin content to about 10% in the treated biomass. Further lignin reduction incurs an extra cost; therefore, it is not justified by increments in glucan conversion [26]. Although the removal of hemicellulose could increase with increasing alkali loading, the glucan conversion did not increase linearly. Moreover, the high removal of xylan was negative to the efficient utility of sweet sorghum stalks. Taking into account solid recoveries and glucan conversion, the optimized alkali loading was determined to be 2.5 mmol of NaOH per gram of dry biomass. Using this alkali loading, 61.66% of lignin was removed from the sweet sorghum bagasse, and a relatively high carbohydrate recovery of 91.56% was achieved. Moreover, NaOH was a better choice and was used in the following experiments due to having a lower price than KOH.

### Distillation combined with NaOH pretreatment operated in a distillation stripper

In our designed process, the distillation of fermented sweet sorghum bagasse with alkali was performed in a distillation stripper. Fermented sweet sorghum bagasse (4 kg wet weight) was blended with 250 mL of 8 mol/L NaOH solution and loaded in the distillation stripper for distillation. The moisture of the mixtures was about 80%. The loading of NaOH was 2.5 mmol/g of dry biomass, which was optimized in the preliminary experiment of distillation combined with alkaline pretreatment. For convenience, this value was converted into 10% (w/w DM) NaOH. Chen *et al*. reported that the enzymatic hydrolysis of carbohydrates substrates correlates better with biomass alkali loading than with alkali solution concentration [26]. The structural carbohydrate conversion increases with increasing alkali loading on dry biomass, whereas no correlation can be established between structural carbohydrates conversion and NaOH solution concentration [25]. This was attributed to the fact that sodium hydroxide was consumed in the pretreatment as a reactant rather than as a catalyst [28]. This revealed that the best loading used in the preliminary experiment was also adapted to the distillation combined with alkaline pretreatment performed in the distillation stripper, although the water content was different (the moisture of the bagasse was 76%). The composition of bagasse following alkaline pretreatment performed in the distillation stripper is shown in Table 3. The carbohydrate content of bagasse, including glucan and xylan, obtained from the distillation stripper was 57.28% and 32.86%, respectively. These were both higher than those of bagasse obtained from the preliminary experiment. In contrast, the lignin content of 6.67% was lower than that in the preliminary experiment. The results demonstrated that using equal alkali loading, pretreatment in the distillation stripper was more efficient than that performed in the flask. This may be attributed to the higher heat-efficiency of steam compared to an electric heater.

**Table 3:** Composition of 10% (w/w dry mass) sodium hydroxide-treated sweet sorghum bagasse with ethanol distillation

**Composition**	**Content (%)**
**Glucan**	57.28 ± 0.42
**Xylan**	32.86 ± 0.64
**Acid soluble lignin (ASL)**	1.13 ± 0.12
**Acid insoluble lignin (AIL)**	5.54 ± 0.22
**Ash**	0.66 ± 0.06

All data are shown as mean ± SD.

### Optimization of enzyme loading

**Figure 6:**
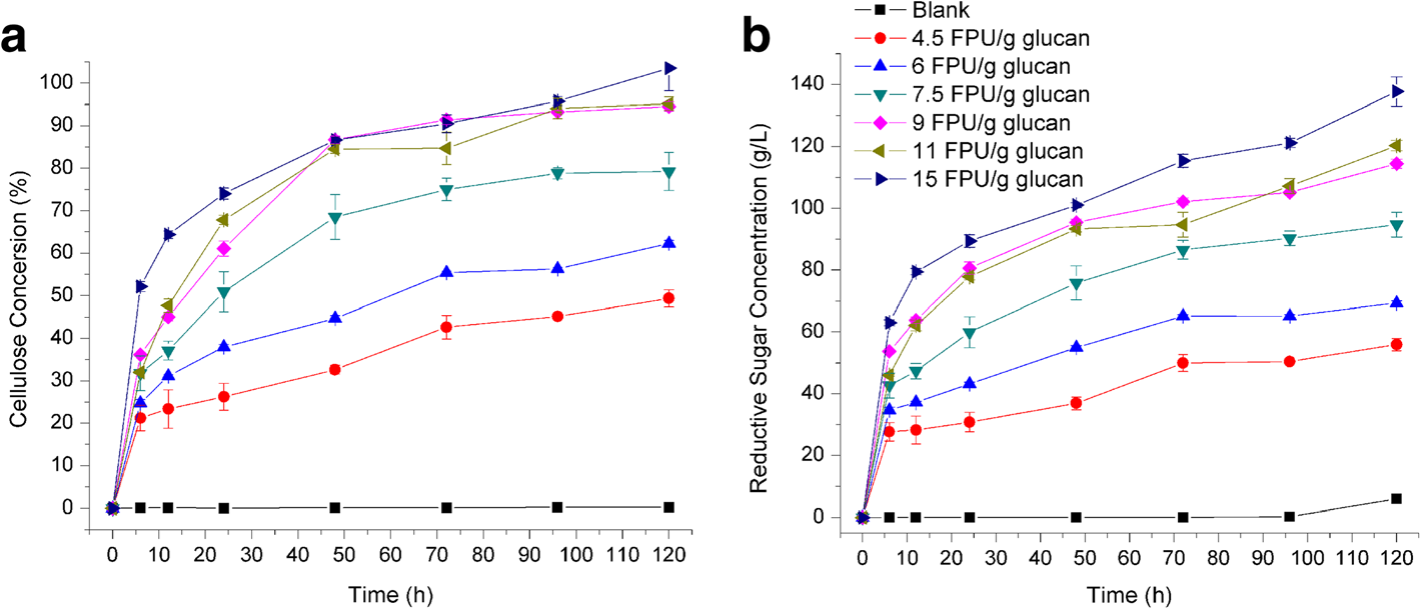
**Enzymatic hydrolysis of sweet sorghum bagasse following various alkali pretreatments operated in a distillation stripper. a**: Cellulose conversion of sweet sorghum bagasse following various alkali pretreatments; **b**: Reduction carbonhydrates concentration of hydrolysed solution of sweet sorghum bagasse following various alkali pretreatments. FPU, filter paper cellulase units.

Commercial cellulose Cellic CTec3 was used in the enzymatic hydrolysis of sweet sorghum bagasse following treatment with 10% (w/w DM) NaOH in a distillation stripper. Enzymatic hydrolysis experiments were performed at solid loading of 15% (w/w) with enzyme doses of 4.5, 6.0, 7.5, 9.0, 11.0, and 15,0 filter paper cellulase units (FPU)/g glucan, respectively. The results are shown in Figure 6. The results indicate that higher glucan conversion was obtained by increasing cellulase dosage up to 9 FPU/g of glucan. With further increasing enzyme loading, glucan conversion did not increase significantly, whereas the enzymatic hydrolysis rate increased continuously. That means more enzyme better decreases the hydrolysis time. Moreover, the speed of glucan conversion increased rapidly in the initial 12 h, and this increment obviously slowed after 48 h. However, to obtain relatively high glucose recovery, the enzymatic hydrolysis would be better carried out for 72 h because 4 to 10% of glucose recovery was still obtained during the last 24 h. Cellic CTec3 is a state-of-the-art cellulase and hemicellulase complex reported by Novozymes, so reducing sugar including glucose and xylose was obtain after enzymatic hydrolysis of sweet sorghum treated with 10% (w/w DM) NaOH. The total reductive sugar concentrations at varying enzyme loading doses are shown in Figure 6b. When the enzyme loading exceeded 9 FPU/g of glucan, the concentration of total reductive sugar was more than 100 g/L for 72 h. The maximum reductive sugar concentration of 137.8 g/L was achieved at an enzyme loading of 15 FPU/g for 120 h. From an economic viewpoint, enzyme loading of 10 FPU/g glucan, and hydrolysis time of 72 h were adopted in our process. Under this condition, the final glucan and xylan conversion was 92.0 and 53.3% respectively, and the final concentration of reductive sugar was 116.9 g/L.

### C5-C6 anaerobic co-fermentation of hydrolysed slurry

Although partial hemicellulose was removed in the distillation with alkali, there was still a considerable amount of hemicellulose left in the residual bagasse. The results show that there was 8.69% of glucose and 2.99% of xylose in the hydrolysed slurry obtained from enzymatic hydrolysis of bagasse. Cost-competitive ethanol yield from lignocellulose requires fermentation of both hexose and pentose constituents [29], so C5-C6 anaerobic co-fermentation was performed with an engineered strain of *Z. mobilis* TSH-01 under the condition optimized by our research team. For 36-h fermentation, a fermentation broth containing 4.3% of ethanol was obtained. The glucose conversion was 95.1% and the xylose conversion was 65.2%. The lower conversion of xylose was attributed to the short fermentation time.

**Figure 7:**
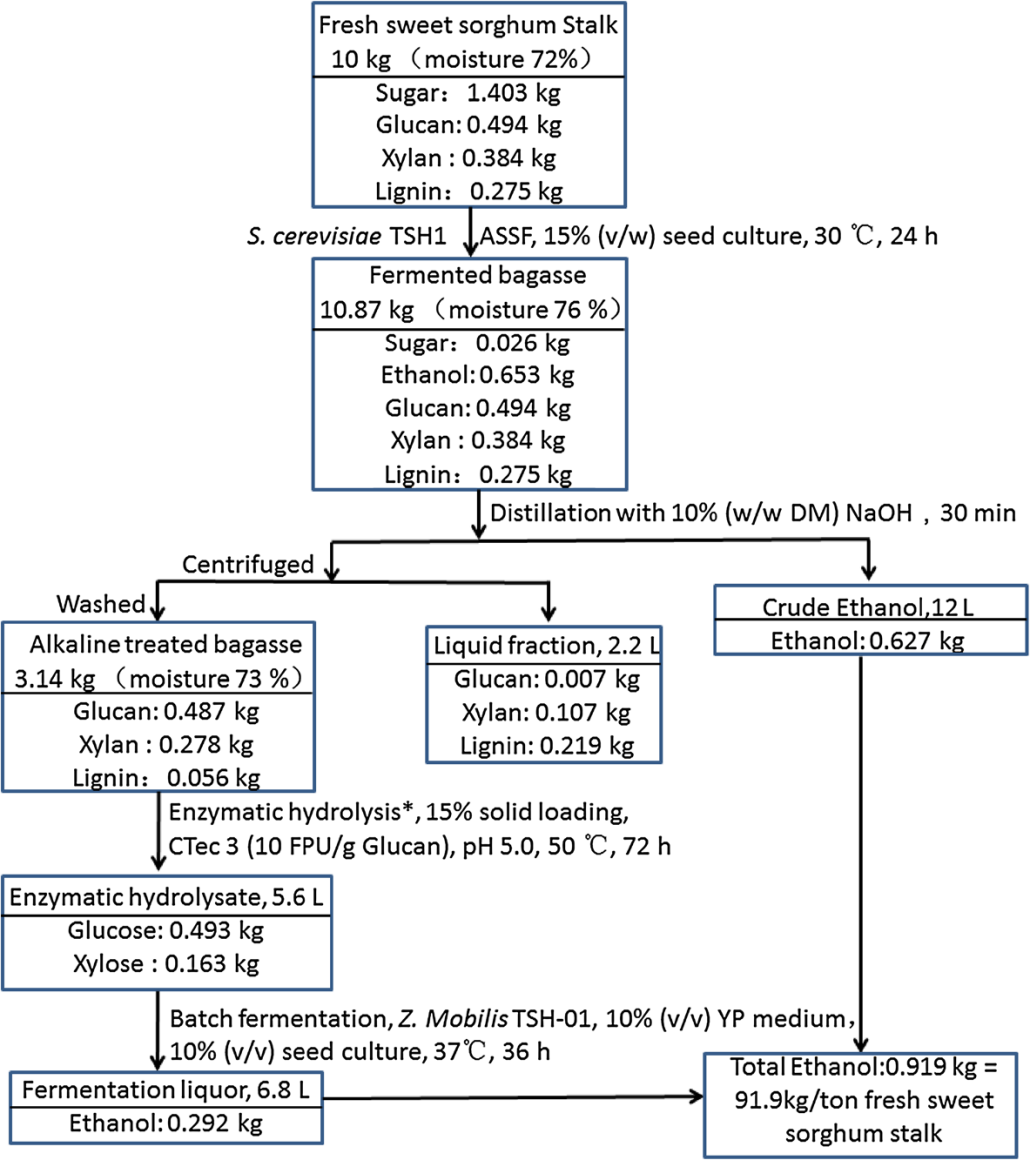
**Mass balances for novel cost-efficient integrated processes for ethanol production from sweet sorghum stalks.** ASSF, advanced solid-state fermentation technology; NaOH, sodium hydroxide; DM, dry mass; FPU, filter paper cellulase unit.

A mass balance starting from 10 kg of fresh sweet sorghum stalks for our overall process for ethanol yield is shown in Figure 7. In the enzymatic hydrolysis and C5-C6 co-fermentation stage, the data were converted according to the results obtained from batch experiments performed in a shake flask instead of a large-scale instrument. It was found that 91.9 kg ethanol/tonne fresh sweet sorghum stalk was obtained, 62.7 kg ethanol from non-structural carbohydrates and 29.2 kg of ethanol from structural carbohydrates. In the solid fermentation step, the ethanol recovery was 87.7%. In the cellulosic ethanol production step, there was 62.0% ethanol recovery due to the highest removal of xylan. The total ethanol recovery of the overall process was 71.55%. Although the ethanol recovery was not high, the ethanol yield was 328 kg ethanol /tonne dry sweet sorghum stalk. This value was higher than the average cellulosic ethanol yield of 300 kg/tonne of biomass [30] and the soluble sugar ethanol yield of 280 kg/tonne [22]. The energy input and output of this novel process was also calculated and the results are shown in Table 4. To produce 1 tonne of ethanol, the energy input in our process was 12,481.2 MJ/tonne, and the energy input in other cellulosic ethanol processes is from 17,430 to 33,330 MJ/tonne [31].

**Table 4:** Energy input and output for novel cost-efficient integrated processes for ethanol production from sweet sorghum stalks

**Process**	**Input (MJ)**		**Output (MJ)**	
Smash	Electricity	327.1		
Preheat	Electricity	80.6		
	Vapor	58.6		
Seed culture	Electricity	147.4		
	Vapor	58.6		
Fermentation	Electricity	209.2		
	Reaction heat	502.0		
Stripper	Electricity	60.9		
	Steam	7,561.3	Ethanol	20,163.0
Certification	Electricity	65.3	Lignin	5,386.5
Enzymatic saccharification and fermentation	Electricity	761.6		
Distillation and separation	Electricity	2,437.1	Ethanol	9,388.5
Other biorefinery^a^		380.8		
Total		12,650.5		34,938.0

Analysis based on 1 tonne 99.5% ethanol. ^a^Included process water, effluent restoration, capital equipment. Based on average of Energy and Resources Group (ERG) Biofuel Analysis Meta-Model (EBAMM) spreadsheet of Farrell [32].

## Conclusion

In the present study, a novel low-energy consumption process for ethanol production involving first and second ethanol production from sweet sorghum was designed based on distillation combined with an alkali pretreatment process. NaOH loading of 10% (w/w DM) was determined as optimum in the pretreatment combined with the distillation step. Enzyme loading of 10 FPU/g of glucan during 72 h was selected for the enzymatic hydrolysis step. Enzyme loading of 10 FPU/g of glucan, and hydrolysis time of 72 h was confirmed in the enzymatic hydrolysis step: 91.9 kg of ethanol/tonne of fresh sweet sorghum stalk was obtained in the present work. Extraction of sweet sorghum juice, which has high energy-consumption, was avoided in our novel process. Energy and time consumption for pretreatment of sweet sorghum bagasse was also avoided by combining the pretreatment step and the first-generation ethanol distillation step in one step in one reactor, so the capital cost for the pretreatment reactor was also saved. This novel process is efficient to reduce the ethanol production cost and implement bioconversion of all carbohydrates in sweet sorghum stalks. Based on this technology, the recalcitrance of lignocellulose was destructed and the biodegradation of lignocellulose into fermentable sugar is feasible. Bioconversion of sweet sorghum bagasse into different product such as biogas, butanol, and chemicals from fermentation of sugar was feasibly performed under low-energy consumption conditions, so it is considered a promising process for a sugar-based lignocellulosic resource, such as sweet sorghum and sugarcane.

## Methods

### Biomass

Sweet sorghum, Chuntian 2#, was harvested in October 2011, in Huanghua country, Hebei province. Leaves and husks were stripped by hand. The stem was crushed into particles of 1 to 2 mm in diameter, and 3 to 20 mm in length by pulverization, and was stored in sealed plastic bags at -20°C. Feedstock composition was determined using the National Renewable Energy Laboratory (NREL) standard Laboratory Analytical Procedures (LAP) for the determination of the composition of biomass [33]. The composition of the fresh stem is listed in Table 5. All chemicals used in the study were reagent grade and used directly from purchase.

**Table 5:** Composition analysis of the sweet sorghum stalk

**Component**	**Wet biomass %**	**Dry biomass %**
Sugar	14.03 ± 0.22	50.10 ± 0.78
Glucan	4.94 ± 0.05	17.64 ± 0.18
Xylan	3.38 ± 0.04	12.07 ± 0.14
Acid soluble lignin	0.91 ± 0.01	3.26 ± 0.05
Insoluble lignin	1.84 ± 0.01	6.56 ± 0.04
Ash	0.09 ± 0.00	0.33 ± 0.02
Others	2.88	10.37

The moisture of the sweet sorghum stalk is 72%. All data are shown as mean ± SD.

### Microorganism

*S. cerevisiae TSH1* was used as the fermentation strain in the solid fermentation step. The microorganism was conserved in yeast extract peptone dextrose (YPD) medium at 4°C (1% yeast extract, 2% peptone, 2% glucose). In order to maintain the viability of the strain, the microorganism was sub-cultured before each experiment. An engineered *Z. Mobilis TSH-01* recombined by Tsinghua University was used as the fermentation strain in the C5-C6 co-fermentation step. The microorganism was conserved in RM culture medium at 4°C (1% yeast extract, 0.2% monosodium phosphate (NaH_2_PO_4_), 2% glucose). In order to maintain the viability of the strain, the microorganism was sub-cultured before each experiment.

### Enzymes

Enzymatic hydrolysis was carried out using the commercial enzyme Cellic CTec2 or Cellic CTec3, both kindly provided by Novozymes investment Co. Ltd (Beijing, China). The enzymatic activity was measured with Whatman No.1 filter paper according to the NREL method [32]. The filter paper enzymatic activity was 113 FPU/mL and 213 FPU/mL for Cellic CTec2 or Cellic CTec3, respectively.

### Advanced solid-state fermentation

Around 10 kg of crushed sweet sorghum was fully blended with 15% (v/w) of TSH1 seed (about 25 g /L, dry weight) and loaded onto 50 L fermenter, 0.7 m in length and 0.3 m in diameter, designed by our laboratory. The fermentation was carried out at 30°C for 24 h with a rotary speed at 0.5 rpm. Samples were collected at the start and end points of fermentation. Ethanol concentration was determined by gas chromatography (GC). Sugar concentration was determined by high-performance liquid chromatography (HPLC).

### Distillation combined with alkaline pretreatment

#### *Preliminary experiments of distillation combined with alkaline pretreatment*

In order to investigate the effect of alkali loading on the pretreatment, a certain amount of fermented sweet sorghum bagasse was mixed with a certain amount of alkali to achieve the expected loading dose according to the values listed in Table 6, and then the mixture was distilled in a 500-mL round-bottom flask for 30 minutes, which started when the first drop of distillate was observed. Then, 100 mL of distillate were collected to determine the ethanol-recovery yield. All the experiments were performed in duplicate. The solid residues were washed with distilled water until the pH value was 7.0 and dried in an oven at 50°C. The dry weight and the composition of the bagasse following alkaline pretreatment were analyzed by the NREL method [33,34].

**Table 6:** The loading dose of different alkalines used in the distillation

**Loading dose (mmol/g dry biomass )**	**NaOH (g)**	**KOH (g)**	**Ca(OH)**_**2 **_**(g)**
0.83	0.50	0.70	0.93
1.67	1.00	1.40	1.85
2.50	1.50	2.10	2.80
3.33	2.00	2.80	3.70
4.17	2.50	3.50	4.60
5.00	3.00	4.20	5.60
6.67	4.00	5.60	7.40

The dry mass of sweet sorghum bagasse is 15 g and the water is 250 g in the distillation experiments. NaOH, sodium hydroxide; KOH, potassium hydroxide; Ca(OH)_2_, calcium hydroxide.

### Distillation combined with NaOH pretreatment performed in a distillation stripper

Around 4 kg of fermented bagasse were mixed completely with 250 mL of NaOH (8 mol/L) concentrated solution, which provided the final alkali loading of 10% (w/w DM). The final moisture content was 76.3%. This mixture was loaded into a 50-L distillation stripper, 0.45 m in height, and 0.4 m in diameter, designed by our laboratory. Then, 0.15 MPa of steam was injected into the distillation stripper. The monitored operating temperature was kept at 100°C during the distillation stage. The operation time was 30 minutes, which started at the moment when the first drop of the distillate was observed. Another 4 kg of fermented bagasse without alkali was distilled as a control. The distillate was collected and ethanol concentration was analyzed by GC.

Following treatment the solid residues were centrifuged to remove the black liquor fraction and were washed by tap water (until the pH was 7.0) as required. The wet solid sample was stored in sealed plastic bags at -20°C. Some of it was dried in an oven at 50°C to determine the moisture and composition.

### Enzymatic hydrolysis

Enzymatic hydrolysis of sweet sorghum bagasse followed the preliminary experiment of distillation combined with alkali pretreatment. Enzymatic hydrolysis of sweet sorghum bagasse following pretreatment was carried out in a 100-mL shake flask, using 50 mM sodium citrate buffer (pH = 5.0) at 50°C and 120 rpm for 72 h. Sodium azide (3 g L-1) was added to inhibit microbial growth: 1 g of dry biomass was added in each flask, and then the buffer solution was added to the final solid concentration of 5% (w/w). Cellulase used in enzymatic hydrolysis was a commercial cellulase mixture, Cellic CTec2 (113 FPU/mL). The cellulase loading was 20 FPU g^-1^ of glucan. After enzymatic hydrolysis, 1 mL of the sample was taken from reaction mixture and centrifuged at 10,000 rpm for 10 minutes. The supernatant was stored at -20°C prior to HPLC analysis of reducing sugar concentration. All the experiments were performed in triplicate. One control experiment without cellulase was carried out to avoid the effect of residual sugars in sweet sorghum bagasse.

### Enzymatic hydrolysis of sweet sorghum bagasse following distillation combined with alkaline pretreatment operated in a distillation stripper

Enzymatic hydrolysis of sweet sorghum bagasse following pretreatment was carried out in a 500-mL shake flask with 20 glass balls (4 mm in diameter), at 50°C and 150 rpm for 120 h. Then, 1 M of sodium citrate buffer solution was added to the flask containing the washed bagasse, and distilled water was added until the final buffer concentration of 50 mM and pH of 5.0 was obtained. The mixture was sterilized in an autoclave at 121°C for 30 minutes. Sterile water was added until the final solid loading was 15% (w/w). The cellulase used in enzymatic hydrolysis was a commercial cellulase mixture, Cellic CTec3 (214 FPU/mL). Aliquots of the enzyme hydrolysates were taken at different time intervals (Figure 5) and the concentration of the reducing sugar in the hydrolysate was measured by HPLC. All the experiments were performed in triplicate. One control experiment without cellulase was carried out to determine the soluble sugars in the pretreated materials. This value as a blank was subtracted from the final sugar concentration after enzymatic hydrolysis, to calculate the glucan conversion yield.

The enzymatic hydrolysis of the washed bagasse, following distillation combined with 10% (w/w DM) NaOH treated in a distillation stripper for C5-C6 co-fermentation, was performed for 72 h. Other conditions were the same as previously mentioned in this section. At end of hydrolysis, 0.5 mL of enzyme hydrolysate was taken out with a sterilized pipette and heated at 95°C for 5 minutes. The concentration of the reducing sugar in the hydrolysate was measured by HPLC. Ten parallel experiments were performed.

The glucan and xylan conversion achieved following enzymatic hydrolysis was calculated according to the following equations:

$$\text{Glucan}\;\text{conversion}\;\left(\%\right)=\frac{\text{Glucose}\;\text{concentration}\;\times \;\text{Volume}}{\text{Glucan}\;\text{content}\;\text{of}\;\text{pretreated}\;\text{bagasse}\times 1.11}\times 100$$

$$\text{Xylan}\;\text{conversion}\;\left(\%\right)=\frac{\text{Xylose}\;\text{concentration}\;\times \;\text{Volume}}{\text{Xylan}\;\text{content}\;\text{of}\;\text{pretreated}\;\text{bagasse}\times 1.14}\times 100$$

Volume is the volume of C5-C6 hydrolysed slurry.

### C5-C6 anaerobic co-fermentation of hydrolysed slurry

As nutrient, 10% (v/v) of concentrated YP (1% yeast extract, 10% peptone) was added to the shake flask containing the hydrolysed slurry, and then the hydrolysed slurry was inoculated with 10% (v/w) of *Z. Mobilis TSH-01* seed (2.5 g/L dry weight). All the fermentations were performed at 37°C, pH 6.0, and 100 rpm for 48 h. Samples were taken at 0 h and 24 h, centrifuged at 15,000 rpm, and 4°C for 10 minutes. The supernatant was stored at -20°C for the sugar and ethanol measurement. Ten parallel experiments were performed.

### Analytical methods

#### *Dry matter*

Percent solids (% TS) measurements were made using a 105°C-oven method according to standard procedures developed at NREL [34].

#### *Sugar*

Sugar concentrations were measured using HPLC (Shimadzu LC-20 AD, Tokyo, Japan) equipped with a column (Bio-Rad HPX-87H, 250 mm × 4.6 mm, Beijing, China) operating at 60°C with a mobile phase of 5 mM sulfuric acid (H_2_SO_4_) aqueous solution with a flow rate of 0.5 mL/minute using a refractive index (RI) detector. Prior to analysis, the samples were diluted with ultrapure water, and then filtered through 0.45 mm filter (Millipore, Beijing, China).

#### *Ethanol*

Ethanol concentrations were determined by a gas chromatography (Shimadzu GC-14C, Japan) equipped with a flame ionization detector. A 0.125-cm I.D., 2 m, SS column was used using nitrogen gas (N_2_) as a carrier gas and hydrogen gas (H_2_) as a flaming gas. The injector temperature was 80°C, and the detector temperature was 220°C. The running time was 18 minutes.

## Abbreviations

AIL: Acid insoluble lignin; ASL: Acid insoluble lignin; ASSF: Advanced solid-state fermentation technology; Ca(OH)2: Calcium hydroxide; DM: Dry mass; EBAMM: Energy and Resources Group (ERG) Biofuel Analysis Meta-Model; FPU: Filter paper cellulase unit; GC: Gas chromatography; HPLC: High performance liquid chromatography; KOH: Potassium hydroxide; LAP: Laboratory Analytical Procedures; NaOH: Sodium hydroxide; NREL: National Renewable Energy Laboratory; RI: Refractive index; S. cerevisiae: *Saccharomyces cerevisiae*; TS: Total solid; WM: Wet mass; YPD: Yeast extract peptone dextrose; Z. mobilis: *Zymomonas mobilis*.

## Competing interests

The authors declare that they have no competing interests.

## Authors’ contributions

JL designed the experiments, conducted the pretreatment and wrote the manuscript. SL (Supervisor) conceived idea and edited the manuscript. BH conducted compositional analysis and enzymatic saccharification. MY and QL conducted ASSF. YJ conducted the carbohydrate analysis. All authors read and approved the final manuscript.
